# Statistical optimization of waste molasses-based exopolysaccharides and self-sustainable bioelectricity production for dual chamber microbial fuel cell by *Bacillus piscis*

**DOI:** 10.1186/s12934-023-02216-w

**Published:** 2023-10-06

**Authors:** Ebtehag A. E. Sakr, Dena Z. Khater, Zeinab M. H. Kheiralla, Kamel M. El‑khatib

**Affiliations:** 1 Botany Department, Faculty of Women for Arts, Science and Education, Ain Shams University, Cairo, Egypt; 2 Chemical Engineering and Pilot Plant Department, National Research Centre (NRC), El Buhouth St., Cairo, 12622 Dokki Egypt

**Keywords:** *Bacillus piscis*, Optimized molasses-based media, EPS, COD removal, Decolorization, Electrochemical activity

## Abstract

**Background:**

The application of exopolysaccharide-producing bacteria (EPS) in dual chamber microbial fuel cells (DCMFC) is critical which can minimize the chemical oxygen demand (COD) of molasses with bioelectricity production. Hence, our study aimed to evaluate the EPS production by the novel strain *Bacillus piscis* by using molasses waste. Therefore, statistical modeling was used to optimize the EPS production. Its structure was characterized by UV, FTIR, NMR, and monosaccharides compositions. Eventually, to highlight *B. piscis'* adaptability in energy applications, bioelectricity production by this organism was studied in the BCMFC fed by an optimized molasses medium.

**Results:**

*B. piscis* OK324045 characterized by 16S rRNA is a potent EPS-forming organism and yielded a 6.42-fold increase upon supplementation of molasses (5%), MgSO_4_ (0.05%), and inoculum size (4%). The novel exopolysaccharide produced by *Bacillus* sp. (EPS-BP5M) was confirmed by the structural analysis. The findings indicated that the MFC's maximum close circuit voltage (CCV) was 265 mV. The strain enhanced the performance of DCMFC achieving maximum power density (PD) of 31.98 mW m^−2^, COD removal rate of 90.91%, and color removal of 27.68%. Furthermore, cyclic voltammetry (CV) revealed that anodic biofilms may directly transfer electrons to anodes without the use of external redox mediators. Additionally, CV measurements made at various sweep scan rates to evaluate the kinetic studies showed that the electron charge transfer was irreversible. The SEM images showed the biofilm growth distributed over the electrode’s surface.

**Conclusions:**

This study offers a novel *B. piscis* strain for EPS-BP5M production, COD removal, decolorization, and electricity generation of the optimized molasses medium in MFCs. The biosynthesis of EPS-BP5M by a *Bacillus piscis* strain and its electrochemical activity has never been documented before. The approach adopted will provide significant benefits to sugar industries by generating bioelectricity using molasses as fuel and providing a viable way to improve molasses wastewater treatment.

## Introduction

**Fig. 1:**
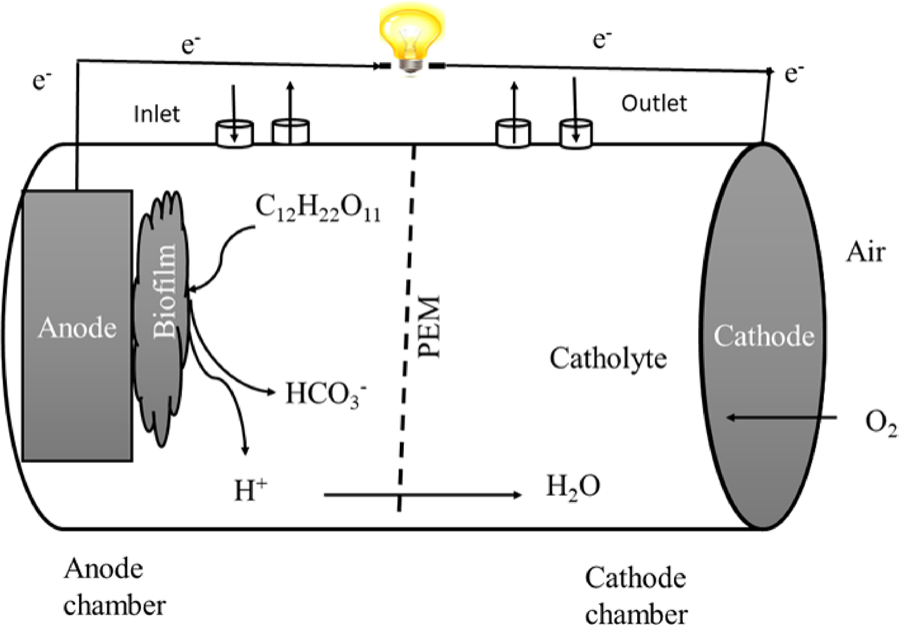
A conceptual view of a dual-chamber MFC experimental system

The most serious issue now plaguing the globe is thought to be climate change. The global depletion of fossil fuels and its detrimental effects on the environment might be seen as the fundamental obstacle to resolving the environmental health issue. Therefore, the search for environmentally friendly technology is viewed as the most promising method of producing sustainable energy [1, 2]. Among renewable energy sources like wind and solar energy, microbial fuel cells (MFCs) are regarded as a new sewage treatment method [3]. MFCs are employed to oxidize different organic substrates through bacterial metabolism in order to produce bioelectricity and eliminate pollutants at the same time [4, 5]. MFCs typically come in one of two designs: a single chamber or two chambers divided by a proton exchange membrane (PEM). The spotlight here is a dual-chamber MFC [6], as illustrated in Fig. 1.

The dual-chamber MFCs are appropriate for anaerobic digestion, bioelectricity generation, wastewater treatment, low energy consumption, low running costs, low sludge production, and clean techniques [7]. Currently, the main factors impeding its advancement are poor power output, high cost, and inadequate wastewater treatment [7]. Electricigens, the MFC's structure, the characteristics of the substrate, external and internal resistances, and the materials used for the electrodes are the foremost issues that have an impact on performance. Simple molecules including sucrose, glucose, fructose, ethanol, and acetate, as well as various wastewater from domestic, industrial, and agricultural sources, can be used as substrates [8]. Several investigations have been done in the past using easily degradable sugars added exogenously to the MFC to generate electricity. However, the performance of the process varies in real-world applications using wastewater depending on the wastes utilized as the substrates and the microorganisms present [9, 10].

Molasses is a syrupy by-product of the sugarcane industry and has a thick, viscous, and dark brown color [11]. Its use as a raw material for the generation of biofuels such as bioethanol, biodiesel, biogas, hydrogen, and methane as well as bio-electricity results in significant amounts of molasses wastewater [12]. Before discharging it into the environment, a suitable wastewater treatment system is needed because dumping it into the water system could result in major pollution, including foul odor and taste as well as effects on the city's water supply and aquatic inhabitants [13]. Some techniques, including anaerobic treatment, adsorption, flocculation precipitation, catalytic oxidation, and membrane separation, are the primary ways for treating molasses wastewater. These techniques share the traits of being expensive and producing harmful byproducts. It is important to look at more suitable treatment options as we deal with the simultaneous challenges of energy shortages and environmental pollution. Due to its poor fluidity, high concentration of organic acids, high color, low pH, and significant corrosivity, molasses has not received much research, however some findings from earlier years have shown that it is an effective electron donor in MFCs [4, 8].

Molasses wastewater contains between 40 and 55% sucrose and has a pH between 5.5 and 6.5. It can be used as a cheap substrate for MFCs because it contains the majority of the nutrients, such as sugar, protein, amino acids, and vitamins, which improve the production of biomass and EPS when used by microbes for the reduction of most molasses into small molecular sugars that are easier for microorganisms to use [14]. According to the literature, total COD removal efficiencies attained with MFC systems have ranged from 45.7 to 90.3%, and the maximum power density (MPD) has been reported to have ranged from 8.8 to 1410.2 mW m^−2^ [15, 16]. For instance, a stacking MFC was created to handle molasses wastewater, and its MPD was 115.5 mW m^−2^ [17].

Microbes linked to MFC produce a thin layer of microbial growth called biofilm that adheres to the electrodes [18]. The location where the biofilm develops controls the bacteria's ability to produce electrons and has a substantial impact on the disintegration of various organic materials in MFC [18]. For the development of biofilms, EPS and lipopolysaccharides are essential. Recent studies have shown evidence that the EPS in electroactive biofilm (EAB), which connects cells to cells over the electrode, is electrically conductive [19]. The synthesis of EPS is often regarded as one of the strategies employed by microorganisms to endure environmental stress [20]. Therefore, EPS producing bacteria was isolated from molasses.

Bacillus sp. has been proven to be a potent source of EPS production [21] and produces more varieties and larger amounts of EPS [22]. Rarely is molasses employed in the production of polysaccharides [23]. Meanwhile, some polysaccharides such as pullulan, welan gum, and glucan have all been produced from molasses [24–26]. For improved EPS production with the development of a less expensive medium, the growth parameters were optimized using Plackett–Burman design (PBD) and statistical design response surface methods (RSM). RSM is one of the effective statistical methods for planning experiments, developing models, identifying the best combinations of variables to produce desired results, and assessing the relative importance of various influencing factors even in the face of complex interactions [27]. One of the most widely utilized experimental models in the RSM for maximizing the production of EPS from *Bacillus licheniformis* NS032 in a medium based on sugar beet molasses is the Box-Behnken Design (BBD) [28]. With this approach, a large number of factors can be optimized simultaneously, and a small number of experimental runs can yield a lot of large quantitative information [29]. An exciting area of research for industrial biotechnologists is the optimization and characterization of innovative EPS-BP5M synthesis from *Bacillus* sp. grown on inexpensive molasses using an ecologically friendly microbial method. It is important to note that the research on EPS-BP5M and bioelectricity production from molasses optimization via this strain has not yet been reported elsewhere.

This study was designed to screen the bacterial isolates from molasses for their ability to synthesize EPS-BP5M and identify the best candidate for bio-based polymer production. The EPS-BP5M yield was optimized by PBD and RSM for the highest EPS-producing bacteria. Then, the structural characterization of EPS-BP5M was done by Fourier-transform infrared (FTIR) analysis, proton nuclear magnetic resonance (^1^H-NMR), elemental analysis, and High-Performance Liquid Chromatography (HPLC). Also, the present study was focused on exploring the EPS producing *Bacillus* sp. for DCMFC electricity production using optimized molasses as a growth medium. Various parameters like OCV, CCV, current density, polarization curve, CE, CV, and its kinetic studies, and anodic biofilm were analyzed by SEM. The removals of COD and color presented in the molasses media were monitored.

## Results

### Chemical analysis of sugarcane molasses

The amount of sugar was found to be fermentable (39.98%) and un-fermentable (4.30%). 48.76% of the total sugar can be regarded as a potential carbon source for many microorganisms. The amounts of residual sugar, inverted sugar, ash content, and pH were 4.21%, 18.60%, 13.10%, and 5.6, respectively (Table 1).

**Table 1:** The physicochemical parameters and EPS yield of bacterial isolates of sugarcane molasses

	Values
*Physicochemical parameters*	
pH	5.60
Total sugar (TS), %	48.76
Total Fermentable sugars (TFS), %	39.98
Non-fermentable sugars (NFS), %	4.30
Residual sugar (RS), %	4.21
Inverted sugar, %	18.60
Ash	13.10
Brix	83.93
*EPS yield (g/L) of bacterial molasses isolates*	
1-Mol	2.94
2-Mol	2.90
3-Mol	2.46
4-Mol	1.26
5-Mol	5.78

### Isolation, selection, and genotypic identification of the potent EPS producing isolate

Five colonies (1-Mol–5-Mol) isolated from molasses were chosen in light of the observation of mucous growth surrounding its colonies on agar plates. The isolate 5-Mol was determined to have the largest EPS production (5.78 g L^−1^ of medium) than the other isolates, making it a promising candidate for EPS production.

**Fig. 2:**
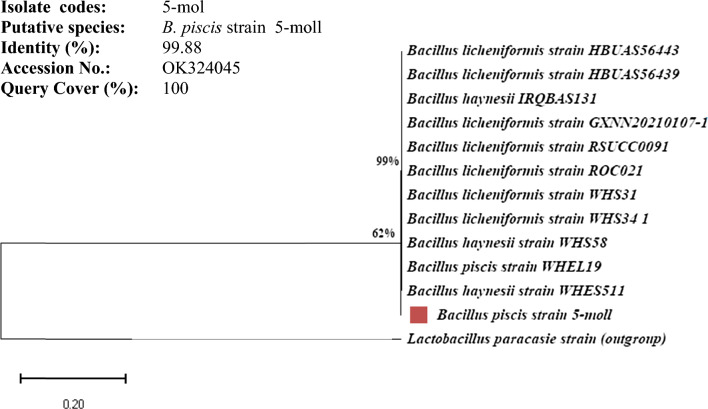
Phylogenetic relationships between 5-mol isolate and 16S rRNA gene sequences retrieved from the GenBank database

The 5-Mol isolate was identified molecularly utilizing the partially sequenced 16S rRNA genes and revealed to be a member of the genus *Bacillus* with a 99.88% identity to the species *B. piscis* with a query cover of 100%. A phylogenetic tree (Fig. 2) based on 16S rRNA gene sequences was created to show the relative positions of this strain (5-Mol) and other *Bacillus* species. The sequence was additionally submitted into the GenBank database (NCBI) under accession number OK324045. The identified strain was designated as *B. piscis* strain 5-mol. *B. piscis* has never before been isolated from molasses.

### Statistical optimization of molasses based-medium for EPS-BP5M production by B. piscis

#### Plackett–Burman design (PBD)

Utilizing a cheap nutrient source like sugarcane molasses (industrial waste) is one of the cost-effective solutions for the growth of *B. piscis*. In this study, the PBD was used in 12 runs to screen a total of 11 factors for their impact on the EPS-BP5M synthesis (Table 2). Bacterial EPS-BP5M varied significantly from 12.12 to 27.50 g L^−1^. This variation demonstrated the importance of optimization for achieving the highest yield of EPS-BP5M. The greatest EPS-BP5M yield (27.5 g L^−1^) was recorded in run No. 1, whereas run No. 4 showed the lowest EPS-BP5M production (12.12 g L^−1^, Table 2).

**Table 2:** Optimization of EPS-BP5M production from *B. piscis* in molasses-based medium by PBD

Run	F1	F2	F3	F4	F 5	F6	F7	F8	F 9	F10	F 11	Response ( EPS-BP5M Yield)	
	A: Molasses, (X1)	B: Yeast ex., (X2)	C: Peptone ex., (X3)	D: NaCl, (X4)	E: KH_2_PO4, (X5)	F: K_2_HPO4, (X6)	G: NaNO_3_, (X7)	H: MgSO_4_, (X8)	J: MgCl, (X9)	K: Inoculum size, (X10)	L: pH, (X11)	Actual value	Predicted value
	%	g/L	g/L	g/L	g/L	g/L	g/L	g/L	g/L	%	pH	g/L	
1	8	2	2	0.5	0.3	1	5.0	0.8	0.5	6	8	27.5	27.26
2	2	2	2	0.5	0.1	1	2.5	0.2	0.5	2	6	14.05	13.86
3	8	4	2	0.5	0.1	2	2.5	0.8	2.5	2	8	23.88	24.20
4	2	2	2	1.0	0.1	2	5.0	0.2	2.5	6	8	12.12	11.68
5	8	4	2	1.0	0.3	2	2.5	0.2	0.5	6	6	24.98	26.00
6	8	2	4	1.0	0.3	1	2.5	0.2	2.5	2	8	18.63	19.07
7	2	4	4	1.0	0.1	1	2.5	0.8	0.5	6	8	17.22	16.95
8	2	4	2	1.0	0.3	1	5.0	0.8	2.5	2	6	14.3	14.06
9	2	2	4	0.5	0.3	2	2.5	0.8	2.5	6	6	16.05	16.99
10	8	2	4	1.0	0.1	2	5.0	0.8	0.5	2	6	25.03	24.51
11	8	4	4	0.5	0.1	1	5.0	0.2	2.5	6	6	26.89	25.87
12	2	4	4	0.5	0.3	2	5.0	0.2	0.5	2	8	13.5	13.69

*F* factor

The first-order polynomial (Eq. 1), which depicts the association between each variable and the EPS-BP5M yield, was constructed from the PBD data as follows:

1 $$Yield\,of\,EPS{ - }BP5M\,\left( {{\text{gL}}^{ - 1} } \right)\, = \, 13.52569 + 1.6575*Sugarcane\,molasses,\,\,\left( {X1} \right) + 0.61583*Yeast\,extract,\,\,\left( {X2} \right) - 3.19667*{\text{NaCl}},\,\,\left( {X4} \right) + 3.83611*{\text{MgSO}}_4 ,\,\,\left( {X8} \right) - 0.8675*{\text{MgCl}},\,\,\left( {X9} \right) + 0.64042*Inoculum\,size,\,\,\left( {X10} \right) - 0.70417*pH,\,\,\left( {X11} \right)$$

Molasses, MgSO_4_, MgCl, and inoculum size were shown to significantly affect the production of EPS-BP5M with *P-*values below the significance level in the statistical analysis using PBD (Table 3), while the remaining components were determined to be insignificant with *P-*values above 0.05 for all of them. F-test was used to determine the significance of the fitting equation. The model was extremely significant (*p* = 0.0009 < 0.01) and the R^2^ was 0.9013 and the adjusted R^2^ was 0.9698, which indicated a good model fit.

**Table 3:** Statistical analysis of the model by PBD for EPS-BP5M yield

Source	Sum of squares	df	Mean square	*F* value	*p* value	
Model	359.48	7	51.35	51.53	0.0009*	Significant
A, (X1)	296.71	1	296.71	297.73	< 0.0001*	
B, (X2)	4.55	1	4.55	4.57	0.0994	
D, (X4)	7.66	1	7.66	7.69	0.0502	
H, (X8)	15.89	1	15.89	15.95	0.0162*	
J, (X9)	9.03	1	9.03	9.06	0.0395*	
K, (X10)	19.69	1	19.69	19.75	0.0113*	
L (X11)	5.95	1	5.95	5.97	0.0709	
Residual	3.99	4	1			
Cor total	363.47	11				
R^2^	0.989					
Adj R^2^	0.9698					
Pred R^2^	0.9013					
Adeq. precision	19.114					

A: molasses; B: yeast ex.; D: NaCl; H: MgSO_4_; J: MgCl; K: Inoculum size; L: pH; DF: degrees of freedom

^*^Significant at *P*<0.05. The rest of other factors were non-significant

**Fig. 3:**
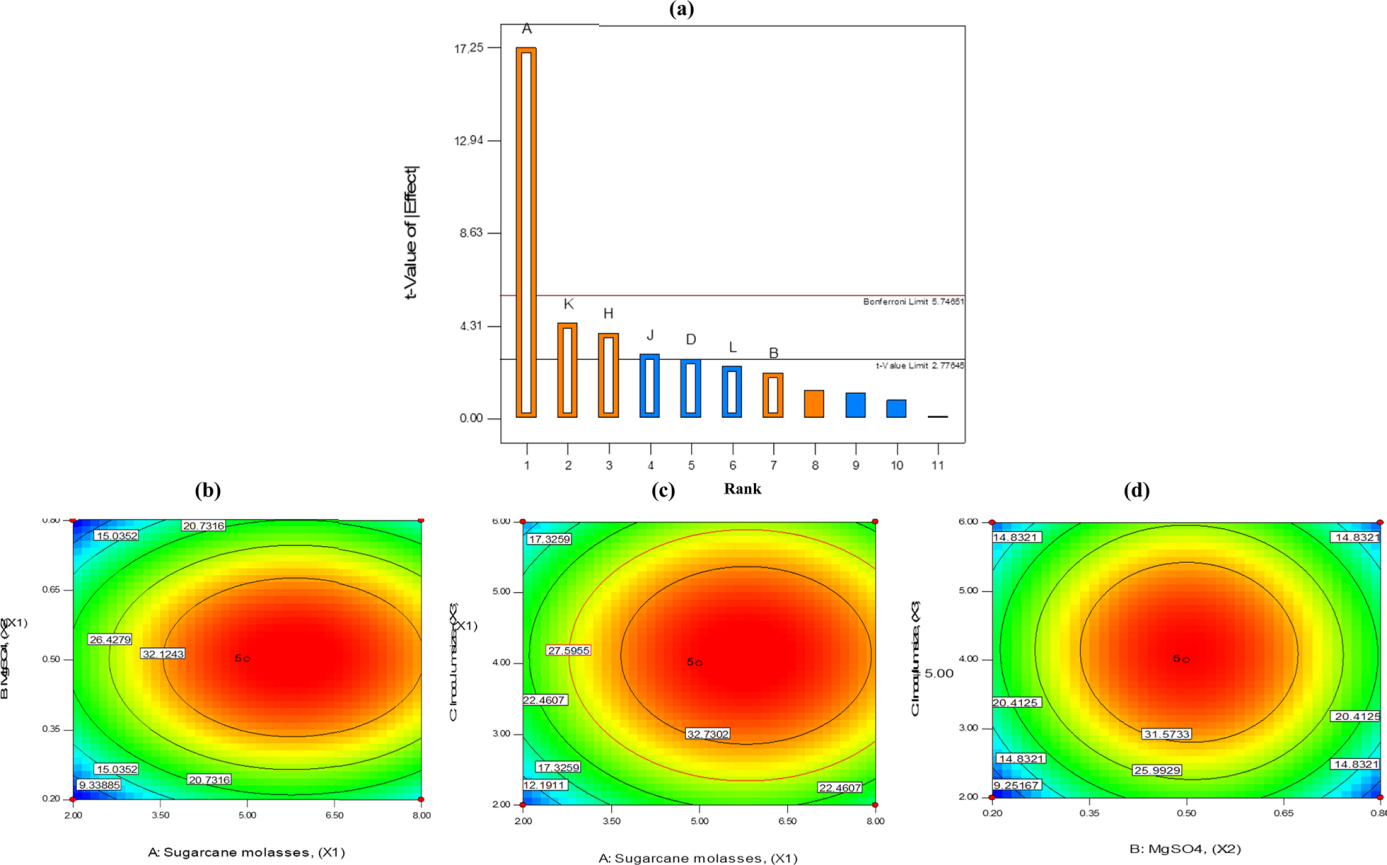
Pareto diagram of the fractional factorial design used to select the variables that affect the production of EPS-BP5M (**a**). Response surface 2D counter plots showing interaction between sugar cane molasses vs. MgSO_4_ (**b**), sugar cane molasses vs. inoculum size (**c**), and MgSO_4_ vs. inoculum size (**d**)

A Pareto chart (Fig. 3) illustrates how the t-value and rankings are related. Depending on the significance level, the Pareto chart indicates the significance and magnitude of the factors that affect the EPS-BP5M production. Effects that exceed the t-value upper limit 2.776 are considered significant. The three variables [molasses (A), MgSO_4_ (K), and inoculum size (H)] were discovered to have a substantial impact on the intended response of EPS-BP5M production based on the effects and *P*-values (Table 3).

#### Box–Behnken design (BBD)

The BBD of RSM was used to ascertain the ideal levels of the three chosen variables (molasses, MgSO_4_, and inoculum size) based on the PBD analysis. Table 4 presents the design matrix and the associated responses. The findings of the experiment were examined using standard ANOVA. The second-order polynomial equation (Eq. 2) was used to fit the BBD:

2 $$Y\,({\text{g}}\,{\text{L}}^{ - 1} ) = 106.66 + 12.90X1 + 201.38X2 + 27.37X3 + 0.28X1X2 + 8.75E - 03X1X3 - 1.14X2X3 - 1.13X1^2 - 196.61X2^2 - 3.26X3^2$$

where Y is the predicted EPS-BP5M production, X1, X2 and X3 corresponded to molasses conc., MgSO_4_ conc_._, and inoculum size, respectively. The proposed model’s F-value was 438.11 and its low *P-*value indicated that it is highly significant according to the statistical analysis performed using ANOVA (Table 5). The model could be used to analyze the variation in the EPS-BP5M yield because the model's R^2^ value was 0.9716 and its adjusted R^2^ value was 0.9959, demonstrating the model's fitting of the tested model and confirming the high accuracy and credibility of the test results [30]. To verify the factors' significance, "Probe > F" was utilized, which denotes how strongly independent components interact with one another. Because each "Probe > F" was less than 0.05, an ANOVA revealed that the model terms X1, X3, X1^2^, X2^2^, and X3^2^ were significant.

**Table 4:** Box–Behnken design for the production of EPS-BP5M

Run	F1	F2	F3	Actual value	Predicted value
	A, molasses (X1) %	B, MgSO_4_ (X2) g/L	C, inoculum size (X3) %	EPS-BP5M Yield (g/L)	
1	8	0.5	2	18.27	17.71
2	8	0.2	4	12.91	13.90
3	5	0.5	4	37.11	37.11
4	5	0.2	2	4.10	3.67
5	5	0.5	4	37.11	37.11
6	8	0.8	4	14.86	15.39
7	8	0.5	6	21.83	20.87
8	2	0.5	6	9.45	10.01
9	5	0.8	6	7.28	7.71
10	5	0.5	4	37.11	37.11
11	2	0.5	2	6.10	7.06
12	5	0.5	4	37.11	37.11
13	2	0.2	4	4.17	3.64
14	2	0.8	4	5.10	4.12
15	5	0.8	2	5.99	6.02
16	5	0.5	4	37.11	37.11
17	5	0.2	6	8.12	8.09

*F* factor

**Table 5:** ANOVA of the different responses assessed for Box-Behnken design for EPS-BP5M

Source	Sum of squares	df	Mean square	*F* value	*p* value	
Model	2993.23	9	332.58	438.11	< 0.0001^*^	Significant
A- (X1)	231.66	1	231.66	305.17	< 0.0001^*^	
B- (X2)	1.93	1	1.93	2.54	0.154	
C- (X3)	18.67	1	18.67	24.59	0.0016^*^	
AB	0.26	1	0.26	0.34	0.5767	
AC	0.011	1	0.011	0.015	0.9075	
BC	1.86	1	1.86	2.45	0.1612	
A^^2^	434.21	1	434.21	571.98	< 0.0001^*^	
B^^2^	1318.37	1	1318.37	1736.68	< 0.0001^*^	
C^^2^	716.24	1	716.24	943.5	< 0.0001^*^	
Residual	5.31	7	0.76			
Lack of Fit	5.31	3	1.77			
Pure Error	0	4	0			
Cor Total	2998.54	16				
R^2^	0.9982					
Adj R^2^	0.9959					
Pred R^2^	0.9716					
Adeq. precision	50.083					

A: molasses; B: MgSO_4_; C: Inoculum size; DF: degrees of freedom

^*^Significant at *P*<0.05

The interactions of molasses, inoculum size, and MgSO_4_ for the production of EPS-BP5M are depicted in a two-dimensional contour graph (Fig. 3). Because the contour plots were elliptical, significant interactions between the inoculum size and molasses and between the inoculum size and MgSO_4_ were also seen.

The regression equation's predicted values were frequently in agreement with the experimental results, demonstrating the model's validity. According to the contour plot and regression analysis, 5% molasses, 0.5 g L^−1^ MgSO_4_, and 4% inoculum size were the ideal conditions to obtain the highest production of EPS-BP5M. The yield was found to be 37.11 g L^−1^ under these optimized conditions with a 6.42-fold enhancement as compared to the initial production medium (5.78 g L^−1^). These findings suggest that EPS-BP5M from *B. piscis* can be produced with a better yield during fermentation by using a waste molasses-based medium as an alternative carbon source.

#### Structural characterization

**Fig. 4:**
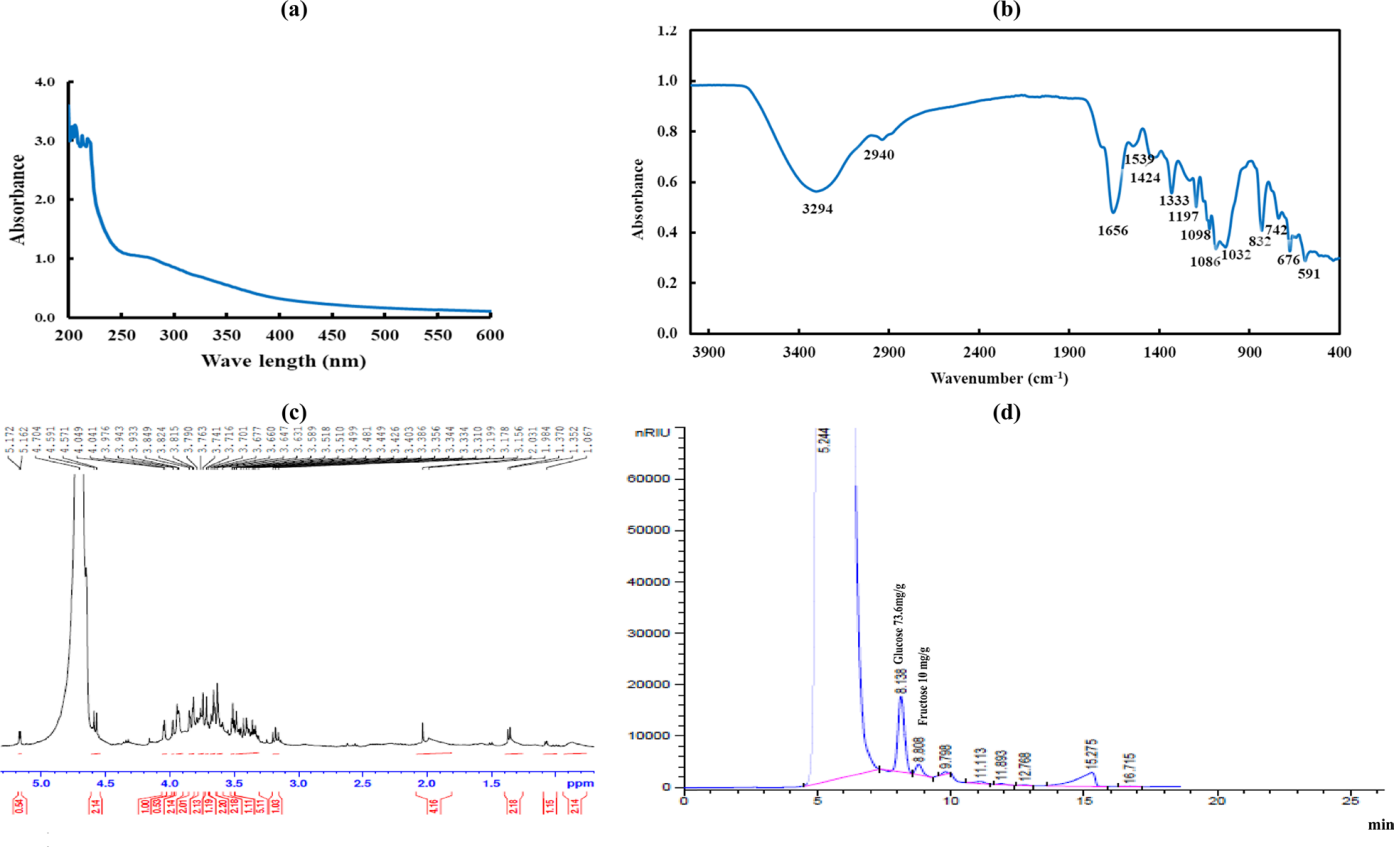
UV (**a**), FTIR (**b**), NMR (**c**) spectra and monosaccharides composition (**d**) of EPS-BP5M from *B. piscis*

No absorption peaks at 260 or 280 nm were detected in the UV–Vis spectra of the EPS-BP5M (Fig. 4a), demonstrating the lack of proteins and nucleic acids [31].

FTIR spectroscopy was used to examine the chemical composition of the EPS-BP5M produced by *B. piscis* (Fig. 4b). The broad stretching peak of O–H stretching is evident at 3294 cm^−1^ [32]. At 2940 cm^−1^, C–H stretching vibration has been found [32]. As a result of C–O stretching, the spectrum data also revealed a peak at 1656 cm^−1^ [33]. Peak between 1000 and 1110 cm^−1^, which is indicative of the presence of α-(1 → 4) Glu*p* residue [34]. There was no peak for the β-configuration, which is expected to rise between 890 and 950 cm^−1^ [34]. The characteristic peaks of carbohydrates were a number of peaks between 1000 and 800 cm^−1^ [35]. Therefore, this molecule might facilitate biofilm formation by *B. piscis* on our MFC.

Most of the proton signals were found between 3.156 and 4.591 ppm in the ^1^H-NMR spectrum of EPS-BP5M (Fig. 4c). Notably, two peaks (5.162 and 5.172 ppm) were found in the anomeric region, indicating the existence of anomeric protons. Hydrogen–deuterium oxide (HDO) is responsible for the peak at 4.70 ppm. Between 4.1 and 3.1 ppm, the frequency of HC–O (singly-oxygenated hydrogen and carbon) chemical shifts was noted [36]. This suggests that EPS-BP5M may have a higher binding capacity to the DCMFC electrode. Therefore, to establish its ability to bind to electrodes, an experiment utilizing *B. piscis* producing EPS-BP5M is required.

The findings of the elemental analysis (Table 6) revealed higher concentrations of carbon and hydrogen, indicating that the majority of the components of EPS-BP5M are sugars.

**Table 6:** Elementary composition of the EPS-BP5M and bacterial surface properties

Parameters	Values
*Elemental composition of EPS-BP5M (%)*	
C	28.56
H	6.68
N	2.89
S	3.41
*Bacterial surface properties*	
Autoaggregation (%)	
3 h	24.12 ± 3.25
6 h	52.67 ± 1.62
24 h	73.48 ± 1.79
Hydrophobicity (%)	31.98 ± 1.12

#### Monosaccharide analysis

The EPS-BP5M was acid hydrolyzed, and its monosaccharide composition was identified (Fig. 4d). The repeating units of this EPS-BP5M were observed by comparing the chromatographic results of the sample with the retention time of various monosaccharide standards. These repeating units were glucose (73.6 mg/g) and fructose (10.0 mg/g). This result implied that strain 5-Mol synthesized a heteropolysaccharide in an optimized molasses medium, with glucose serving as the major repeating monomer in this polysaccharide chain.

#### Cell surface characteristics

*Bacillus* sp. had hydrophobic cell surfaces and the cell autoaggregation improved over time (31.98 ± 1.12%; Table 6). The results clearly showed that EPS-BP5M alters the physicochemical characteristics of cell surfaces, indicating that hydrophobic interaction may be crucial for *B. piscis* cells' ability to adhere to carbon-felt electrode of our DCMFC.

### Utilization of optimized molasses-based medium for electricity production using *B. piscis* strain

#### Voltage generation

**Fig. 5:**
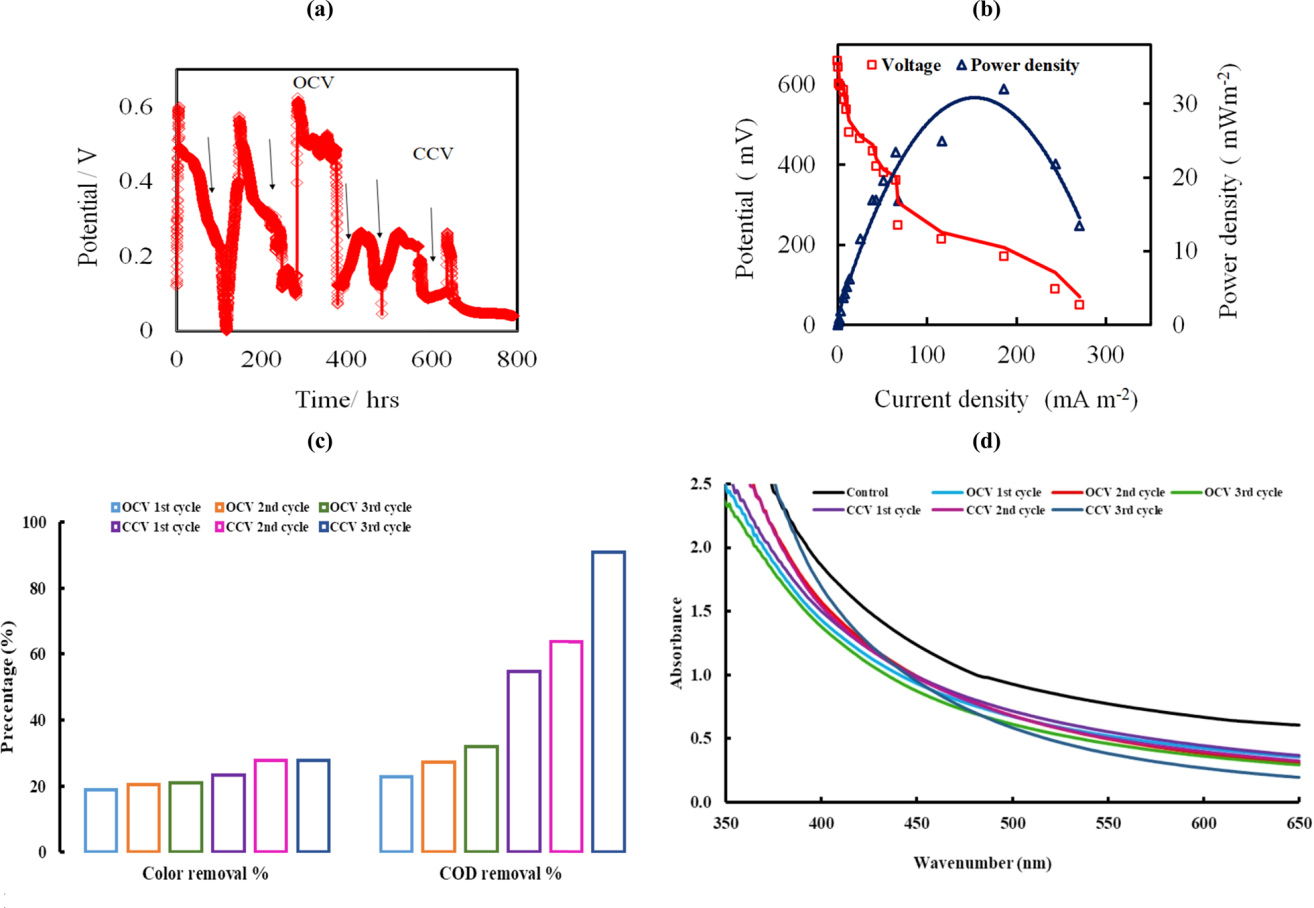
The performance of DCMFC inoculated with *B. piscis* and molasses wastewater as anode fuel, **a** Trend of both OPVs and CCVs output values in the 800 h incubation period and **b** Power and polarization curve. **c** Color (ξcolor %) and COD removal efficiency of DCMFC after each cycle. **d** Spectral scanning (visible band) of optimized molasses media before and after MFC treatment at 37 °C, following inoculation with *B. piscis*

Figure 5a depicts the changes in open circuit voltages (OCVs) and close circuit voltages (CCVs) at an external resistance of 10 kΩ over time following feeding with an optimized molasses-based medium inoculated with *B. piscis*. Our strain had three phases (log, stationary, and decline phase) throughout the acclimatization period when grown on molasses over three cycles. During the log stage, each OCVs cycle started to climb linearly. Throughout 200 h, the voltage outputs gradually stabilized at maximum levels of 574, 599, and 624 mV, respectively, with very minor changes for each cycle. After the OCVs dropped to extremely low values (about 70 mV) due to the loss of fuel cell life, fresh molasses wastewater was added. In such instances, the bacterial communities in the functioning biological reactor already adjusted well to the environment of DCMFC and subsequently takes shorter periods to decrease the startup time for electricity generation [37]. Moreover, Fig. 5a illustrated the influence of 10KΩ on the performance of DCMFC outputs using molasses as an electron donor over three cycles of operation. It could be observed that the CCVs had the same previously pattern.

#### Polarization characteristics DCMFC

To ensure the accuracy of the measurement, several external loads ranging from 650 kΩ to 100 Ω were applied when the OCV reached a plateau after feeding with molasses to determine the representative steady-state polarization properties and the accompanying power density plots for DCMFC. The maximum power density (PD_max_) was observed to be 31.98 mW m^−2^ when the current density (CD) was 185.95 mA m^−2^ and the resistance was 500 Ω at a corresponding voltage output of 172 mV (Fig. 5b). The calculated internal resistance (R_in_) was found to be approximately 463.4 Ω. Molasses contained roughly 50% sucrose by weight, which was thought to be the primary substrate for the DCMFC. The equation for the oxidation of sucrose was determined using a stoichiometric method similar to that used for the oxidation of acetate [38].

The anodic reaction is then given as follows:

3 $${\text{C}}_{12} {\text{H}}_{22} {\text{O}}_{11} + 25{\text{H}}_2 {\text{O }}\mathop{\longrightarrow}\limits^{B. \, piscis}12{\text{HCO}}_3^- + 60{\text{H}}^+ + 48e^-$$

whereas, the cathodic reaction as follows:

4 $${\text{O}}_2 + 4{\text{H}} + + 4e^- \to \, 2{\text{H}}_2 {\text{O}}$$

The polarization curve could be separated into three zones. At the high cell voltage zone, the current increased rapidly with the lowering of the cell voltage values, but at the intermediate voltage zone, the rate of this increase was greatly diminished. At the low cell voltage zone, the rate of the increased current became high again.

#### COD removal efficiency

At the end of each cycle in the DCMFC system, the variation affinity of COD and its removal rate of the molasses wastewater implemented as the anode substrate medium were assessed (Fig. 5c) at an initial COD concentration value was 22 g L^−1^. With a removal efficiency of 25 ± 3.2%, the COD averaged 16.5 g L^−1^ during the OCV cycle. While with a removal efficiency of 59 ± 6.4%, the COD averaged 9 g L^−1^ during the CCV cycle. Moreover, the maximum CCV at 265 mV was obtained for 8 and 2 g L^−1^ while the minimum CCV at 10 g L^−1^ was about 262 mV. As a result, the performance was due to the breakdown of molasses by electroactive *B. piscis,* resulting in a lower COD value.

#### Coulombic efficiencies

The oxidation of molasses over three cycles of operations causes a flow of electrons by mature *B. piscis* biofilm at the anode chamber, which is represented by the coulombic efficiencies (CEs%). It was calculated by monitoring the variations in COD during a period following the cell's voltage reduction to less than 70 mV. The CEs were 36.94, 33.22 and 21.23% at 12, 14 g L^−1^ and 20 g L^−1^ COD, respectively. Additionally, the CE decreased as influent COD molasses concentration increased. It could be confirmed the inverse relationship between C_E_% and molasses concentration.

#### Color removal

After DCMFC treatment, a decolorization of the molasses was observed with a decolorization of 27.68 ± 1.52% (Fig. 5c, d).

#### Cyclic voltammetry (CV)

**Fig. 6:**
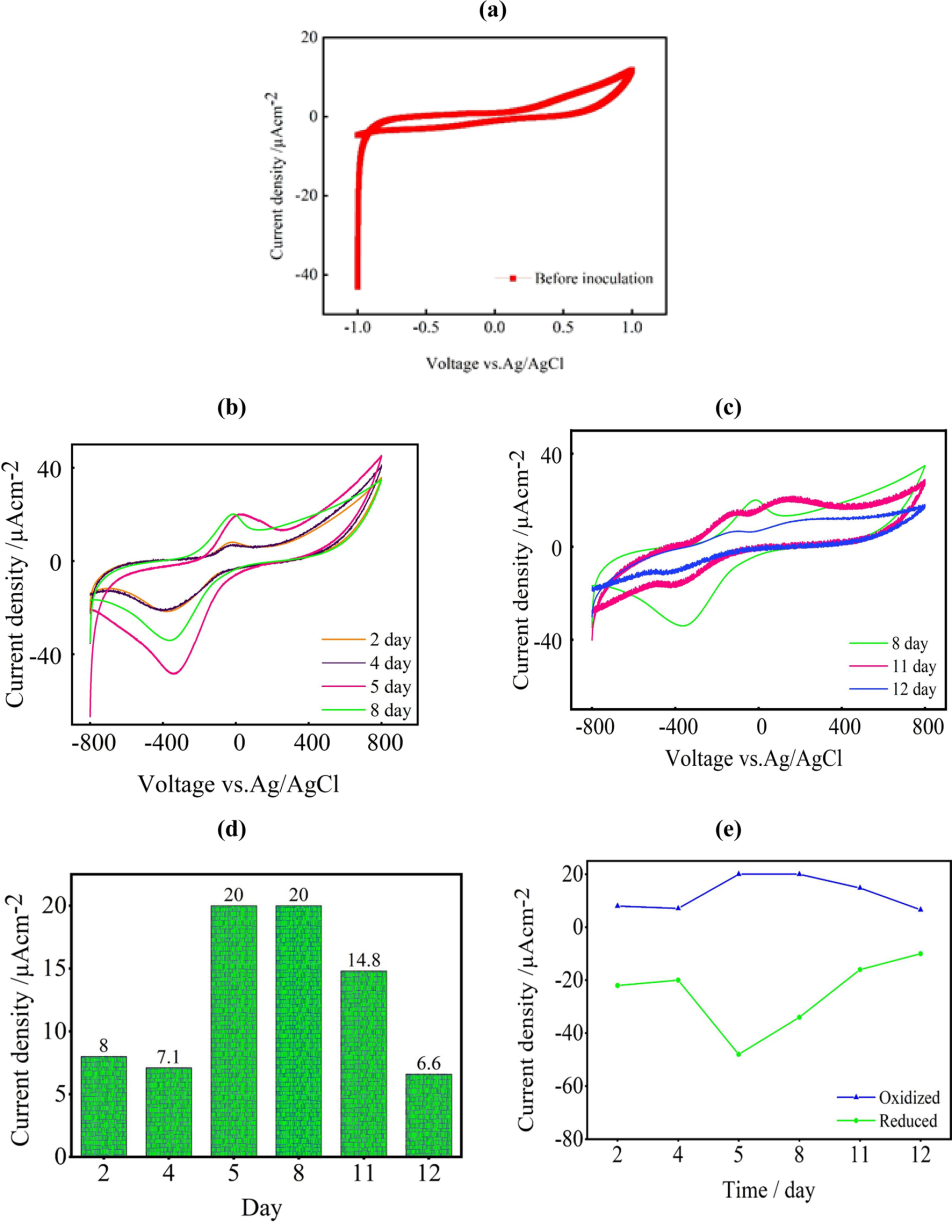
The electrocatalytic activity of sterilized *B. piscis* in an anodic chamber for DCMFC using molasses as substrate, **a** CV before inoculation, **b**, **c** recorded CVs at 20 mV s^−1^ for 2, 4, 5, 8, 11, 12 days, **d** the corresponding current density in correlation with day and **e** the trend of oxidized and reduced peaks over the days

The electrocatalytic activity of *B. piscis* biomass that had been connected to the anodic electrode at various times throughout the operation was confirmed by the CV analysis. Thus, in order to determine the oxidation reduction activities and the mediators linked to the anodic chamber, a sterilized molasses media inoculated with *B. piscis* was incubated at various time intervals (0 h and 2, 4, 5, 8, 11, and 12 days) at a scan rate of 20 mV s^−1^ (Fig. 6).

The CV displayed a smooth curve prior to inoculation (0 h) with no redox peaks during the oxidation and reduction process (Fig. 6a). While the *B. piscis* displayed two reversible peaks, as shown in Fig. 6b, c, which point to the existence of redox peaks in the molasses-based media. Additionally, Fig. 6d, e depicts the trend of both oxidized and reduced peaks over the days and the produced current densities. The voltage and current of oxidized and reduced peaks were significantly shifted along the operation time, which could be related to alterations in the medium over time. Furthermore, redox peaks started to form at − 24 mV after 2 days of inoculation. The oxidation and reduction peaks then started to rise at − 26 mV after 4 days of incubation and ultimately reached their peak after 5 days. The highest current densities were recorded on days 5 and 8 of operation, measuring at 20.08 and 20.06 µA cm^−2^ with corresponding positive sweep potentials of 25 and 3.5 mV, respectively.

In addition, it was noted that the 11 day (− 125 and − 153 mV) and 12 day of operation (− 140 and 210 mV) had two oxidative waves. Reduction peaks were seen in the potential range between − 103 and − 720 mV during the reverse scan. The released extracellular metabolites produced by *B. piscis* and the self-produced mediators that formed during the late exponential and stationary phases may be responsible for these waves [39]. After 12 days, the peak current dropped to 6.6 µA cm^−2^ at − 105 mV, presumably due to nutrient depletion, as no medium refill was done [40–42]. These findings match with the cell proliferation displayed in Fig. 5a. The cell growth peaked after 5 days of operation and entered the exponential phase. These results encourage further study into whether *B. piscis* acts well as a biocatalyst for the breakdown of molasses in MFCs, taking into account the potential existence of EPS-BP5M which might accelerate the electron transfer.

#### CV studies at different scanning rates

**Fig.7:**
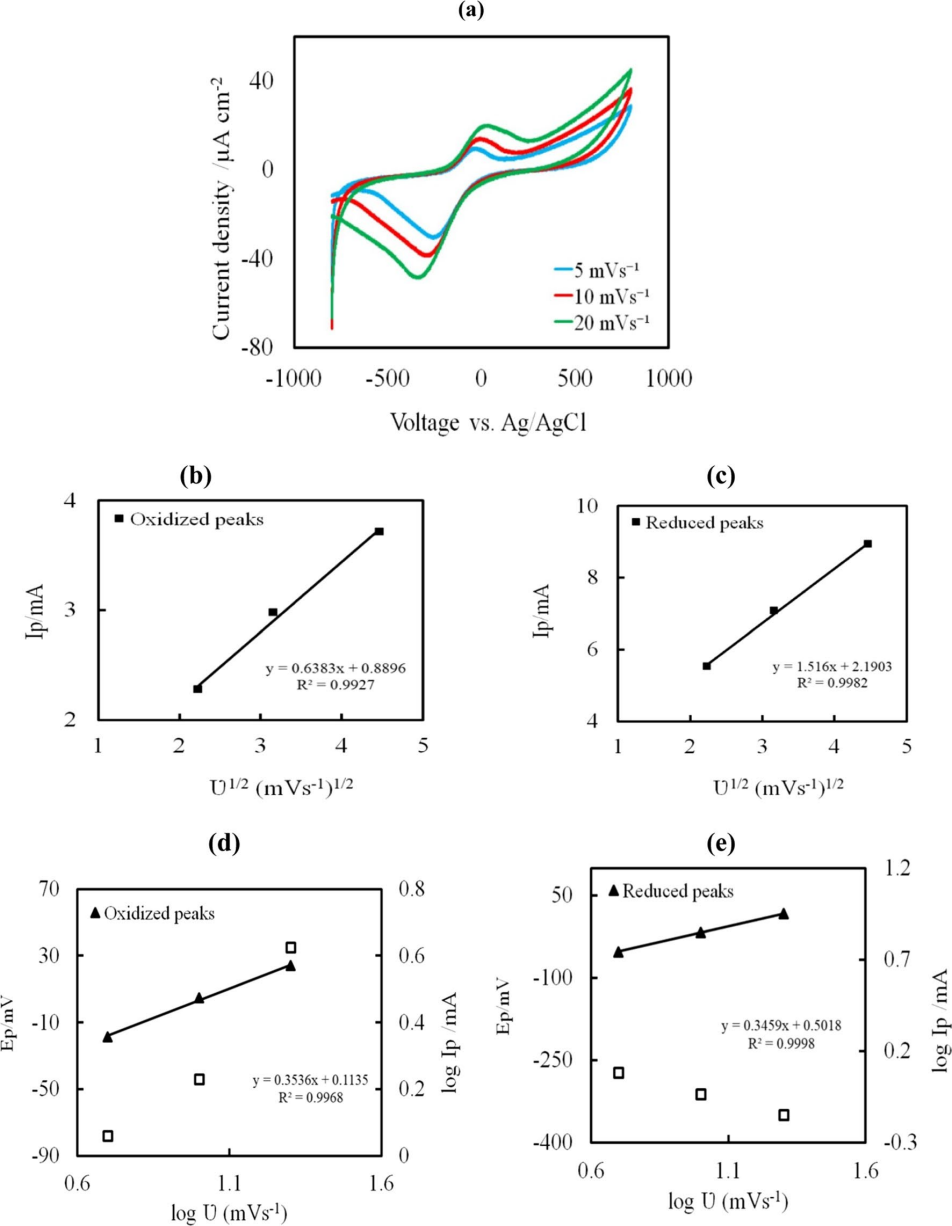
Kinetic study of *B. piscis* oxidation and reduction peaks at the 5th day of inoculation at the scan rates of 5, 10, 20 mV s.^−1^, **a** CV at different scanning rates, **b**, **c** Ip at each redox vs square root of ʋ (Randles–Sevcik equation), **d**, **e** Ep vs log ʋ (plain diamonds) and log I vs log ʋ (black triangles). Ep (peak potential); Ip (peak current); ʋ (scan rate)

On the 5th day, as shown in Fig. 7a, the CV was measured at various sweep scan rates of 5, 10, and 20 mV s^−1^ to assess the kinetic studies on the inoculated DCMFC with *B. piscis* in the anodic chamber. The increase in scan rate was accompanied by an increase in redox peaks. Additionally, the reduction peaks were modified towards a negative voltage value whereas the oxidized peaks were shifted towards a more positive voltage value. Furthermore, only the major peak of oxidation and reduction, which was assumed to be the only simple electrode reaction, was studied. The direct electron transfer generation that was accomplished by the *B. piscis* may be the cause of the increase in peak value with scan rate. Also, the relationship between peak current (Ip) and the square root of scan rates (ʋ^1/2^) for oxidation and reduction peaks was used to calculate the Randles–Sevcik equation (Fig. 7b, c). It was found that the Ip and the ʋ^1/2^ had a linear relationship. Moreover, the charge transfer at the electrode happens more quickly than the active species diffuse from the bulk solution to the electrode surface. Since, there were narrow, symmetrical faradaic peaks in in CV, the process might have been quick and reversible. The relationship between the peak potential (Ep) and scan rate (log ʋ) is seen in Fig. 7d, e. The slope values for the log Ip and log ʋ for the oxidized and reduced peaks were nearly equal (0.35). When *B. piscis* was used as the source of the adsorption processes, the adsorbed nutrients also appeared on the anodic electrode surface. These findings demonstrated that the combination of diffusion and adsorption processes limited the kinetics, demonstrating an irreversible electron charge transfer nature of these peaks [43].

#### Biofilm characteristic

**Fig. 8:**
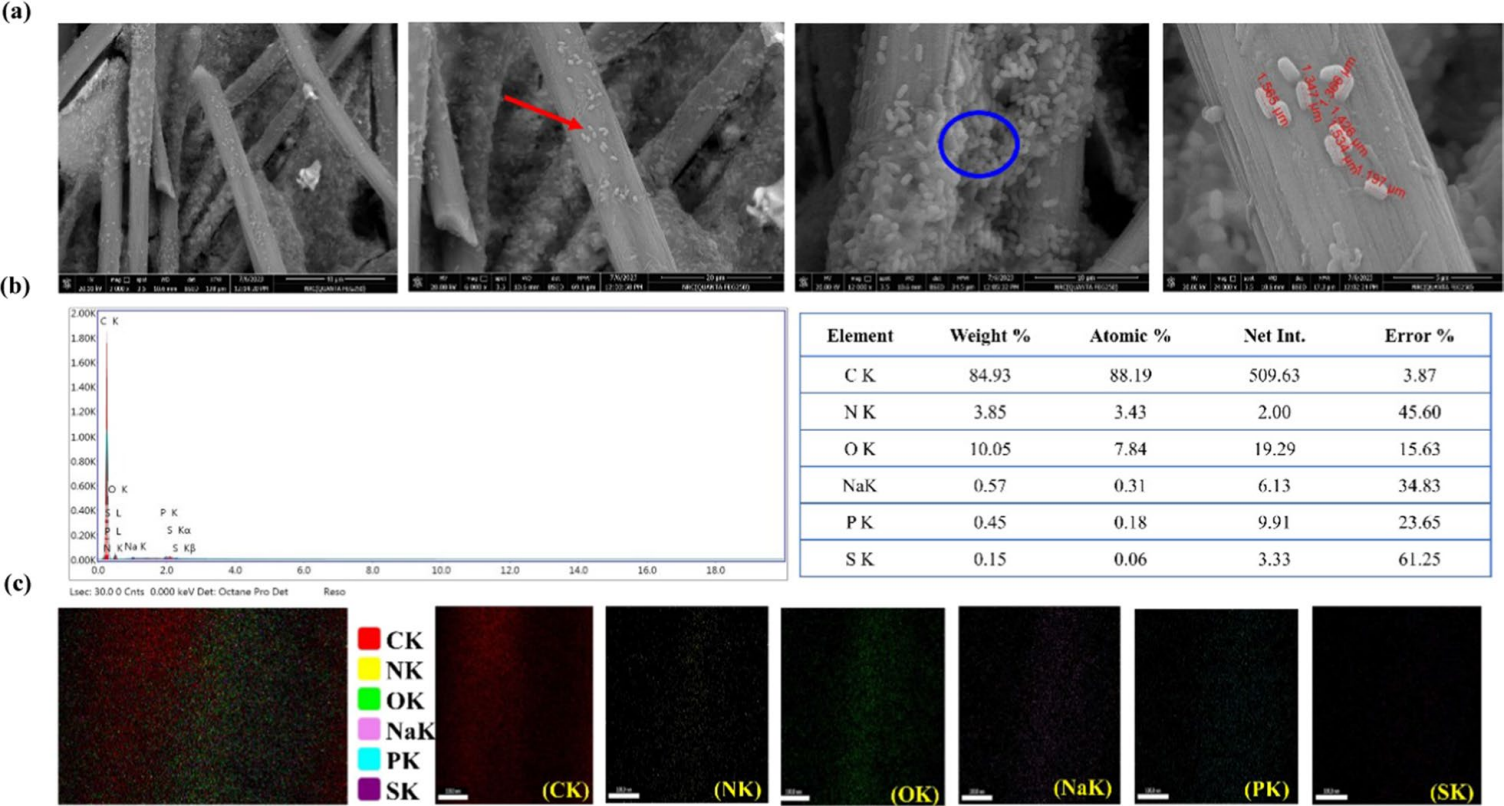
SEM images taken at different magnifications, bacterial cells (red arrow), bacterial attached to electrode fiber forming aggregates (blue circle) (**a**); EDX analysis (**b**); and elemental mapping (**c**) of biofilm formed on carbon felt anode used in the DCMFC for molasses treatment and electrical power generation

After the DCMFC operation, the electrode's surface was examined using SEM. A dense biofilm can easily form in the gaps or holes between the electrode's fibers (Fig. 8a). *B. piscis* was rod cells with a cell length ranging between 1.197 and 1.565 µm (Fig. 8a). The bacteria produced aggregates and gathered in groups, supporting the auto-aggregation of the cells following treatment. This suggests that the aggregates are trapped in the fibrils, where they eventually attach and grow. Bacterial adhesion and colonization on the electrode surface would benefit from the mature EPS-based anodic biofilm.

The findings of the EDX elemental mapping displayed in Fig. 8b demonstrated the presence of the elements C, N, O, Na, P, and S. Figure 8c presents the homogeneous distribution of basic elements. According to an elemental analysis, the anodic biofilm contains a higher carbon content than any other element. The coexistence of EPS-BP5M on the bioanode is confirmed by both EDX and elemental mapping.

## Discussion

Microbial fuel cells (MFC’s) are gaining popularity owing to their eco-friendly and energy generation with bioremediation. Molasses are being explored as alternative low-cost nutrients to produce EPS-BP5M by *B. piscis* isolated from it (molasses). This study examined the efficiency of *B. piscis* in generating electricity and bioremediate the organic matter from molasses wastewater as anode substrate in DCMFC. To our knowledge, only two papers identified *B. piscis* strain. It was isolated from a *Dissostichus mawsoni* muscle sample from the Antarctic [44] and Indian salterns [45]. Different species of *Bacillus* have been found in the wastes of the sugar industry [46, 47].

Any microbial strain can provide greater yields, but the key is to develop the production medium [48]. Several factors affected the production of EPS including microbial species, culture conditions, and nutritional types [49]. Molasses are useful as a growth medium because they contain high levels of vitamins and minerals and have a strong growth-stimulating impact [50]. It has been utilized as a substrate for the fermentation synthesis of biopolymers because of its various benefits, including high sucrose and other nutrient levels, cheap cost, easy availability, and simplicity of storage [51]. After optimizing the process parameters, the maximum EPS-BP5M production was 27.5 g L^−1^ which is higher than the bacterial EPS gained prior to the use of PBD (5.78 g L^−1^) by 4.76 times. RSM has been demonstrated to be a useful technique for evaluating the impact of factors on the production of EPS by *B. licheniformis* NS032 [28], *Leuconostoc citreum* B-2 [52], and *Pantoea* sp. BCCS 001 GH [31] using molasses as substrate.

A high concentration of minerals and vitamins in molasses, in addition to the sugars that are easily fermented, may be the cause of this increase in productivity [53]. The inhibiting effect of high quantities of mineral components would be mitigated by the diluting of molasses and nitrogen limitation can stimulate the formation of polysaccharides [28]. Bacillus strains that produce EPS are more resilient to environmental stress ([54].

Our findings supported previous findings indicating the carbon source had a significant impact on the formation of EPS [55–57]. The growth of microorganisms and the production of EPS require a carbon source as a substrate. Usually, EPS formation is encouraged by a high concentration of a carbon source [58]. Due to its high sucrose content, molasses was found to have a considerable impact on the EPS-BP5M yield during the screening of growth factors. Bacterial cells might use the additional carbon supply to expand quickly and make more EPS at low concentrations of molasses, but at larger concentrations, catabolite suppression of oxidative pathways will occur, slowing down bacterial development [53, 59], because of an increase in osmotic pressure in the medium, which led to plasmolysis and cell death [53]. Our results indicated that the EPS-BP5M yield from *B. piscis* is higher than those of other microorganisms such as *Bacillus subtilis* (4.92 g L^−1^) [53], *Bacillus licheniformis* ANT 179 (16.35 g L^−1^) [60], *B. licheniformis* mutant strain (9.0 g L^−1^) [54], and *Pantoea* sp. BCCS001GH (9.9 g L^−1^) [31].

Auto-aggregation is associated with the development of biofilms [61], which would result if this also happened within our DCMFC. EPS promotes cell aggregation through complicated interactions and also accelerates the development of biofilms and granulation [62]. In the field of wastewater treatment, the ability of microbial cells to auto-aggregate is intriguing as this may encourage the production of bioaggregates and control the efficacy of treatment [63, 64].

*Bacillus* sp. is simple to grow and produces EPS with a high potential for bioremediation [59]. *B. piscis* was well adapted on the surface of the carbon felt electrode, generating a mature electrogenic biofilm, according to stable voltage outputs [3, 17]. The power density for the present study was found to be comparable with many of the reported literature values as shown in Table 7. The PD_max_ values were higher than those examined by Fan et al. (2016). They utilized a DCMFC fueled with simulated molasses wastewater as the anode substrate and showed PD_max_ generation of 31.37 mW m^−2^ with a matching voltage output of about 7.5 mV [7]. Additionally, Lee et al. (2016) reported in another investigation that the DCMFC's highest CD was 80 mA m^−2^ and PD was 17 mW m^−2^ [65]. In contrast, our finding was lower than the research conducted by Syafitri et al. (2018). They reported that the DCMFC produced the maximum voltage (0.372 V) and PD_max_ (813.191 mW m^−2^) when sediment was mixed with molasses [66]. The discrepancy in power density values revealed in various publications may be due to variations in substrates, microbial species, MFC configuration, electrode materials, and PEMs. Our novel *B. piscis* exhibits good performance on electricity generation using molasses wastewater as anode fuel.

**Table 7:** Comparisons of current study performance and other similar studies inoculated with molasses wastewater as substrate

MFC construction	Microorganisms used	OCV (mV)	CCV (mV)	Power density	COD %	CE%	References
DCMFC	*Bacillus piscis*	624	265	185.95 mW m^−2^	90.9	36.9	Current study
H-shape DCMFC	*Meyerozyma guilliermondii*	NA	350	61.25 mW m^−2^	NA	NA	[1]
SCMFC	Sludge	NA	500	169.86 mW m^−2^	NA	13.3	[4]
DCMFC	Sludge	NA	7.5	31.37 mW m^−2^	35.5	NA	[7]
U-shaped DCMFC	Sewage	569	~ 351	88.9 mW m^−2^	NA	81	[8]
DCMFC	*Brevibacillus borstelensis*	990	453	188.5 mW m^−2^	81.7	59.8	[12]
Baffled stacking MFC	Sludge	690		115*.*5 mW m^−2^	70	1	[17]
DCMFC	Yeast	NA	372	813.19 mW m^−2^	97	NA	[62]
SCMFC	*Candida boidinii*	953		5.45W cm^−2^	NA	NA	[64]
DCMFC	*Pseudomonas Sp*	NA	670	660.82 mW m^−2^	NA	NA	[72]
DCMFC	Sludge	NA	392	14.9 mW m^−2^	31.8	6.2	[73]

*SCMFC* single chamber MFC, *DCMFC* dual chamber MFC, *NA* not applied

As the COD concentrations decreased, the overall COD removal efficiency increased yields by 54.55, 63.64, and 90.91%, respectively. These findings suggested that the *B. piscis* activity is more potent and may be employed for COD elimination and voltage generation from molasses [3, 67]. The fermentation mechanisms, biomass formation, and consumption of coulombs during methanogenesis, which take place in the anode chamber through the breakdown of a portion of molasses, could be the cause of the reduction of CE in the MFC [68].

Molasses wastewater has a dark brown color due to the presence of amounts of melanoidins, caramel, and phenolic pigments present. Melanoidins are the hardest to get rid of because it has the largest content and most complex structure of all of them [69]. After DCMFC treatment, a decolorization rate can break down complex compounds like melanoidoin, producing colorless intermediate metabolites [70]. The rates of decolorization in the current study are higher than those previously reported by Mohanakrishna et al. [71], who reported 22.92% of decolorization following MFC treatment of distillery effluent inoculated with mixed consortia.

*B. piscis*' electrocatalytic activity is supported to regulate subsequent oxidation processes and intermediate breakdown [12]. The linear relationship of Ip and the ʋ^1/2^ indicated the performance was subjected to a diffusion-controlled process [72]. Excellent and efficient adsorption occurs through the electrode holes because it enhances the contact between pollutants and exoelectrogens [73]. Bacterial adhesion and colonization on the electrode surface would benefit from the mature EPS-based anodic biofilm. Consequently, it might be said that the bacteria were metabolically active and increased power output [74].

## Conclusions

In the present study, a novel EPS-producing bacteria, *B. piscis*, was isolated and identified from molasses waste. The efficient EPS-BP5M production in an optimized medium with inexpensive sugarcane molasses substrate was also carried out which resulted in higher EPS-BP5M production. To stick to the carbon felt electrode of the DCMFC, the bacterium most likely generates EPS-BP5M. This work offered new insights on the use of bacteria that produce EPS using molasses. EPS-BP5M was characterized using FT-IR, NMR, and HPLC. *B. piscis* could be used as an anodic biocatalyst for the treatment of sugar industry molasses used as a substrate in a DCMFC and simultaneous energy recovery. Our findings show an effective and eco-friendly approach (DCMFC) that uses molasses, a sustainable agricultural by-product, to solve the problem of eliminating organic matter and producing bioelectricity.. Further work will be done on molasses wastewater treatment.

## Material and methods

### Collection and analysis of sugarcane molasses

The sugar company for integrated industries, located in Hawamdiya, Giza, Egypt, kindly donated the sugarcane molasses. The collected material was carried to the lab with care, stored immediately at 4 °C, and then brought to room temperature before usage. According to the Association of Official Analytical Chemists (AOAC) guidelines, the chemical composition was examined [75].

### Isolation of EPS producing bacteria

First, Erlenmeyer flasks holding 25 mL of liquid nutrient broth (NB) medium were inoculated with 1.0 mL of sugarcane molasses and incubated at 37 °C for 48 h. Then, 1.0 mL of that culture was transported to petri dishes with nutrient agar medium amended with 1% sucrose and incubated for 48 h at 37 °C. Striking was used to separate the mucoid colonies into pure cultures, which were then frozen at − 20 °C in NB medium with 25% glycerol.

### EPS production, extraction, and purification

Bacterial isolates were screened for their ability to produce EPS by inoculation of NB medium supplemented with 1% sucrose. Then, the inoculated flasks were incubated at 37 °C for 48 h. The fermentation broth was centrifuged at 6000 rpm for 10 min, and the supernatant was then mixed with Savage reagent (chloroform: *n*-butanol, 2:1 v/v). The organic layer was recovered, combined at a ratio of 1:3 (v/v) with cold absolute ethanol, and then left to stand at 4 °C overnight. The precipitate was collected and mixed with ultrapure water to create a crude EPS solution. This solution underwent a 72-h dialyze in deionized water to produce an EPS. For the following trials, the isolate that produced the highest yield of EPS was chosen.

### Molecular analysis of the selected EPS producing isolate

Using 16S rRNA gene sequencing, the chosen bacteria were identified. Using universal 16S rDNA primers, the extracted and purified DNA was amplified. The amplified product was sequenced and deposited into the NCBI database. The bacterial sequence was submitted to the GenBank database and the accession number was obtained. The isolated bacteria were then aligned, and MEGA software version 11 was used to create phylogenetic trees using the neighbor joining method.

### Molasses-based media optimization

#### Pretreatment of molasses

Molasses used as a carbon source were mixed with distilled water containing 2% sodium dihydrogen phosphate (1:1). The solution was autoclaved at 121 °C for 10 min before being allowed to cool for 24 h. Clarified molasses was the only source of carbon used [76].

#### Fermentation media

During the fermentation procedure, a 250 mL flask was filled with 100 mL of medium. The production medium's composition varied depending on the experimental approach outlined below. Depending on the experimental design, the best isolate (50 × 10^6^ CFU/mL) was employed to inoculate the sterilized medium. The cultivation process took place at 37 °C for 48 h. In order to calculate the yield of EPS-BP5M (g L^−1^) at the end of the fermentation, samples from the liquid culture were taken.

#### Experimental designs

PBD was used to organize 11 independent variables at two levels with 12 runs to rank the impact of various factors on EPS-BP5M yield (Table 2). The tested variables were molasses, yeast extract, peptone extract, NaCl, KH_2_PO_4_, K_2_HPO_4_, NaNO_3_, MgSO_4_, MgCl_2_, inoculum size, and pH.

According to the PBD experiment, molasses, MgSO_4_, and inoculum size were further optimized using the RSM. As shown in Table 4, variables were performed at three-level (low, middle, and high) trials. Design-Expert software created a 17-run experimental design scheme in accordance with the coding design, utilizing the yield of EPS-BP5M (Y) as the response value at the end of fermentation.

Design-Expert 7.0 software was used to output the results of variance analysis of the PBD experiment and Box–Behnken experiment and carry out regression analysis of the results of the Box–Behnken experiment to simulate the prediction equation.

#### Structural characterization

Ultraviolet–visible (UV–Vis) spectroscopy of the sample was evaluated using a Shimadzu UV-1800 UV spectrophotometer with a wavelength range of 200–600 nm. Using an FTIR (Bruker Alpha 11), the EPS-BP5M sample's functional group content was initially validated. One mg of the sample was combined directly with KBr and then quantified using a spectrum. EPS-BP5M's. ^1^H-NMR spectrum was obtained utilizing a BRUKER 400 MHz spectrometer at 25 °C. Following that, the material was dissolved in D_2_O at a 50 mg/mL concentration. Parts per million (ppm) were used to describe chemical changes. The EURO EA Elemental Analyzer was used to conduct an elemental analysis on the carbon, hydrogen, nitrogen, and sulphur weight percentages of EPS-BP5M. Approximately 1.0 mg of the material was burned. Gas chromatography is used to quantify the combustion byproducts (C, H, N, and S), and the ratio of the original sample's constituents is then calculated.

#### Monosaccharide composition

The monosaccharide compositions were analyzed by HPLC using a Shim-pack SCR-101N column with a mobile phase of ultrapure water. A paste was made by carefully adding 0.5 ml of ice-cold 80% H_2_SO_4_ to the EPS-BP5M sample. After that, the paste was carefully mixed for 15 h at room temperature. The paste was then diluted with a solution of ice and distilled water (up to 6.5 mL) until the sulfuric acid strength reached 2N. The solution was further hydrolyzed for 6 h in a sealed tube over a boiling water bath. The hydrolyzate that was produced was neutralized by BaCO_3_ before being filtered and thoroughly rinsed with water. After that, a cation exchange resin (Amberlite IR-120 (H +)) was used to treat the filtrate and washings. A flow rate of 0.7 ml/min was used to analyze the monosaccharide content. After integrating the relevant areas and comparing the results to standard curves made from glucose, fructose, sucrose, and arabinose (Sigma), each carbohydrate concentration was calculated.

#### Bacterial surface properties

Three subcultures of the isolate were performed in NB medium at 37 °C. After centrifuging the active cultures for 5 min at 6000 rpm, they were washed with sterile saline solution. The washed pellets were combined with sterilized phosphate-buffered saline (PBS) buffer for autoaggregation [77] or saline solution for hydrophobicity [78], and the OD_600_ nm was adjusted (A_0_, H_0_). For the auto-aggregation assay, the bacterial culture (8 mL) was incubated at 37 °C, and the auto-aggregation values were recorded at 3, 6, and 24 h (A_t_). It was determined using the equation (%) = 100 * [1 − (A_t_/A_0_)]. To determine the hydrophobicity of the cell surface, hexadecane was employed as a solvent. The cell suspension was mixed with hexadecane, and the process was vortexed for 1.0 min. A_t_ 600 nm (H_1_), the optical density of the aqueous phase was determined after 15 min of separation. The equation affinity (%) = 100 * [1 − (H_1_/H_0_)] was then used to compute the percentage of cells that were transported to the hexadecane phase.

### Synergistic interactions of DCMFC and microbially induced removal of color and COD of molasses waste as the substrate

#### DCMFC construction and operation

The experimental system was mainly made up of a dual-chamber MFC reactor that was assembled by joining two cylindrical plexiglass chambers that served as the anodic and cathodic chambers. These chambers were 6 cm long, 4.6 cm in diameter, and had a total working volume of 100 mL. Proton exchange membrane (PEM, Nafion 117, Dupont Co.), which assures the transmission of only hydrogen ions and no other ions, was used as the separator between the anode and cathode. The anode comprised an unmodified sheet of three-dimensional carbon felt joined to the top of an externally connected anode port with affective dimensions of 2.5 × 2.5 × 0.6 cm and a projected surface area of 18.50 cm^2^. The cathodic electrode was made of non-waterproof gas diffusion carbon cloth with a microporous sheet (6 × 6 cm each; surface area equivalent to 16.63 cm^2^). A stainless steel wire was used to link the electrodes in bioreactors so that electrons could be transferred. A 50 mM phosphate buffer solution was employed in the cathode chamber, which was left open to the atmosphere for ventilation of O_2_ as an electron acceptor, while the anode chamber was completely sealed with epoxy sealant to maintain an anaerobic environment [42, 79]. The anode substrate was obtained in fed-batch mode using the molasses wastewater (g L^−1^) with 50 mL as the carbon source, 2.0 g yeast extract, 2.0 g peptone, 0.5 g NaCl, 0.1 g KH_2_PO_4_, 1.0 g K_2_HPO_4_, 2.5 g NaNO_3_, 0.5 g MgSO_4_, 0.5 g MgCl_2_, and 4% inoculum size. The electrolyte was adjusted to pH 7 with NaOH. *B. piscis* that had first been activated overnight in NB medium was inoculated into DCMFC to aid in the breakdown of molasses. The strain was cultivated in 100 mL of NB medium for 24 h at 37 °C, followed by a 10-min centrifugation at 6000 rpm at 4 °C. Phosphate-buffered saline (PBS) was used to wash the cell pellets before they were adjusted to 50 × 10^6^ CFU/mL. After the sugarcane molasses was consumed and the decline phase, fresh *B. piscis* and molasses were added to the anodic. The experiments were run at room temperature.

#### DCMFC analysis and calculations

The voltage of the DCMFC was recorded every 5 min using a data acquisition system (Lab jack U6-PRO) connected to a laptop, and Ohm’s Law (V = IR) was used to determine the current value when the external resistance was set to 10 kΩ. Open circuit voltage (OCV), which was obtained in the steady state when the circuit was opened. Polarization and power curves were generated by manually stepping down the external resistance from 650,000 to 100 Ω. The current and power was normalized to the anode surface area to calculate the current density (mA m^−2^) and power density (mW m^−2^). Internal resistance is determined by the slope of the linear region of the current–voltage graph. According to APHA standard procedures, COD concentrations in influent and effluent were evaluated. Organic concentrations were estimated as COD removal efficiency (COD R%), which was calculated using the following equation: COD R % = 100 * [(COD_initial_ − COD_final_)/COD_initial_], where COD_initial_ is the COD concentration in the influent (mg COD/L), and COD_final_ is the COD concentration in the final effluent at the end of DCMFC batch cycles (mg COD/L) [4]. The coulombic efficiency (C_E_) expresses the charge efficiency in which electrons were being transferred in the DCMFC. It was calculated by dividing the generated current to the theoretical current as CE = [*M*∫*I.dt* / *nFv*ΔCOD] * 100, where I is current output (A), v is the working volume (L) in the anode, n is the number of electrons exchanged per mole of O_2_ = 4, F is Faraday’s constant (96,485 A s mol^−1^), ΔCOD is change in chemical oxygen demand (g L^−1^), M the molar weight of oxygen [8].

#### Color removal

After DCMFC treatment, molasses samples were diluted 1:10 in triplicate, the pH was set to 7.0, and they were then subjected to a spectral scan by the UV-1800 spectrophotometer from wavelengths between 350 and 650 nm to assess the decolorization. The formula used to determine color removal efficiency (ξcolor %) at 475 nm (the wavelength at which melanoidins absorb best) is ξcolor % = 100 * [1 − (A_i_/A_f_)], where A_i_ and A_f_ refer to initial and final absorbance, respectively. The samples were taken after each cycle to measure the color [70].

#### The electrochemical measurements

Cyclic voltammetry (CV) of sterilized DCMFC that inoculated with a single culture of *B. piscis* (cell suspension of 50 × 10^6^ CFU mL^−1^ at logarithmic phase) was achieved by applying various scan rates at 5, 10, and 20 m mV s^−1^ at different testing periods from 24 to 288 h after cell operation. The CVs were collected using the Voltamaster 6 potentiostat (PST006) in the potential range of − 0.8 to 0.8 V vs. Ag/AgCl. The anode, cathode, and Ag/AgCl (Metrohm) were implemented as working, counter, and reference electrodes, respectively. The study of the reversibility of the electron transfer was depicted by the peak potential dependence on scan rate and linearity of the Randles–Sevcik equation (linear plot of peak current dependence on the square root of scan rate) [80].

#### Biofilm characterization

Using scanning electron microscopy (SEM Quanta FEG 250 with field emission gun, FEI Company, Netherlands), the surface morphologies and microstructures of the anodic biofilm were studied. The biofilm was performed by fixing in 0.1 M phosphate buffer (pH 7.0) containing 2.5% (v/v) glutaraldehyde for an overnight period [81]. The increasing gradients of ethanol (25, 50, 70, and 100%) were used to induce dehydration. The biofilm was allowed to dry at room temperature before being sputter-coated with gold and subjected to analysis at a voltage 20 kV. Additionally, energy-dispersive X-ray spectroscopy (EDX) was used to determine the element content and elemental distribution maps.

### Statistical analysis

The statistical analysis was done using the Microsoft Excel 2016 version. Data analysis dealt with all variables, computations, means, and standard deviations.

## Data Availability

All data generated or analyzed during this study are included in this article.
